# Deep focus light-field camera for handheld 3D intraoral scanning using crosstalk-free solid immersion microlens arrays

**DOI:** 10.1063/5.0155862

**Published:** 2023-08-28

**Authors:** Jae-Myeong Kwon, Sang-In Bae, Taehan Kim, Jeong Kun Kim, Ki-Hun Jeong

**Affiliations:** 1 Department of Bio and Brain Engineering, Korea Advanced Institute of Science and Technology (KAIST), 291 Daehak-ro, Yuseong-gu, Daejeon 34141, Republic of Korea; 2 KAIST Institute for Health Science and Technology (KIHST), KAIST, 291 Daehak-ro, Yuseong-gu, Daejeon 34141, Republic of Korea; 3 Vatech Co. Ltd., 13 Samsung-ro 2-gil, Hwaseong-si, Gyeonggi-do 18449, Republic of Korea

## Abstract

3D *in vivo* imaging techniques facilitate disease tracking and treatment, but bulky configurations and motion artifacts limit practical clinical applications. Compact light-field cameras with microlens arrays offer a feasible option for rapid volumetric imaging, yet their utilization in clinical practice necessitates an increased depth-of-field for handheld operation. Here, we report deep focus light-field camera (DF-LFC) with crosstalk-free solid immersion microlens arrays (siMLAs), allowing large depth-of-field and high-resolution imaging for handheld 3D intraoral scanning. The siMLAs consist of thin PDMS-coated microlens arrays and a metal–insulator–metal absorber to extend the focal length with low optical crosstalk and specular reflection. The experimental results show that the immersion of MLAs in PDMS increases the focal length by a factor of 2.7 and the transmittance by 5.6%–27%. Unlike conventional MLAs, the siMLAs exhibit exceptionally high *f*-numbers up to *f*/6, resulting in a large depth-of-field for light-field imaging. The siMLAs were fully integrated into an intraoral scanner to reconstruct a 3D dental phantom with a distance measurement error of 82 ± 41 *μ*m during handheld operation. The DF-LFC offers a new direction not only for digital dental impressions with high accuracy, simplified workflow, reduced waste, and digital compatibility but also for assorted clinical endoscopy and microscopy.

## I. INTRODUCTION

Volumetric information on tissues plays an important role in tracking and treating diseases.^1,2^ Optical 3D imaging techniques, such as stereoscopy, structured illumination, or confocal scanning, are actively utilized for *in vivo* imaging applications in clinical endoscopy and intraoral scanning.^3–5^ The depth information improves diagnostic accuracy, treatment time, and patient comfort during clinical operations, yet such 3D *in vivo* imaging systems often feature bulky configurations and motion artifacts due to an additional camera, pattern projector, or scanning elements.^3,6^ Recently, light-field cameras (LFCs) employing microlens arrays (MLAs) gain much attention as a new 3D imaging tool due to the simple configuration and the single-shot volume acquisition.^7,8^ However, conventional LFCs still require the extension of the depth-of-field (DoF) for motion artifact-free and facile handheld operation in practical 3D *in vivo* imaging applications.^9^

Large DoF in LFCs requires high *f*-number MLAs matching with a main lens.^10^ In contrast to conventional methods, such as micromolding,^11,12^ microdispensing,^13,14^ and laser manufacturing,^15,16^ MLAs are often microfabricated by using a resist reflow method and recently incorporated with extra features, such as anti-reflective structures^17^ for less specular reflection and optical crosstalk blocking^18,19^ for high-contrast imaging. Resist-reflowed MLAs intrinsically exhibit the *f*-number less than *f*/2.5 due to gravity-induced sagging,^20^ whereas additional features increase the reconstruction accuracy of light-field imaging. Such technical issues hamper the development of large DoF LFCs because the *f*-number of MLAs is matched with the main lens in order to fully ensure the effective area of active pixels on the image sensor.^21^ Multi-focal^22,23^ or tunable^24,25^ MLAs extend the DoF by capturing light-field images with multiple focal lengths. However, they still have some critical drawbacks, such as shallow DoF in a single exposure and limited resolution, which make them unsuitable for handheld operation.

**FIG. 1.**
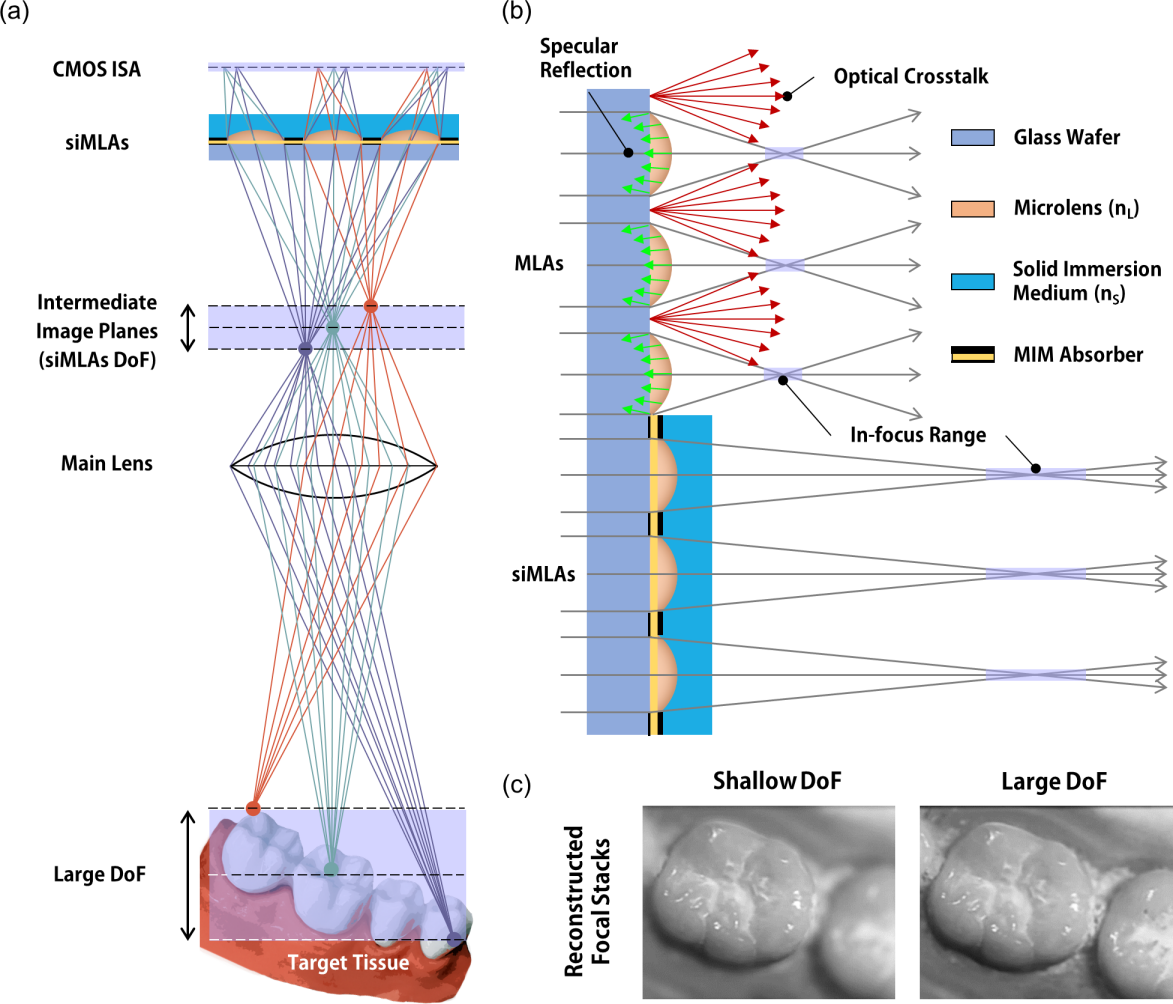
Working principle of deep focus light-field imaging for handheld 3D intraoral scanning. (a) Deep focus light-field camera (DF-LFC) comprising a main lens, solid immersion microlens arrays (siMLAs), and CMOS image sensor arrays. (b) Crosstalk-free siMLAs with high *f*-number. siMLAs substantially increase the focal length without changing the microlens diameter, thereby achieving large depth-of-field (DoF) light-field imaging for facile handheld operation. The metal–insulator–metal (MIM) absorber removes optical crosstalk between microlenses in the visible range to increase the image contrast. (c) DF-LFC captures not only large volumetric information for handheld operation but also high-contrast reconstructed images.

Here, we report a deep focus light-field camera (DF-LFC) for handheld 3D intraoral scanning based on crosstalk-free solid immersion microlens arrays (siMLAs) [Fig. 1(a)]. The DF-LFC consists of a main lens, siMLAs with high *f*-number, and complementary metal–oxide–semiconductor (CMOS) image sensor arrays. Deep focus light-field images are captured by increasing the tolerance for the image plane of the siMLAs, which is also conjugated to the focal plane of the main lens for 3D objects with a large axial variation. The siMLAs employ a thin polydimethylsiloxane (PDMS) layer as a low-index solid immersion medium and a metal–insulator–metal (MIM) absorber between adjacent microlenses [Fig. 1(b)]. Unlike conventional resist-reflowed MLAs, this multi-layered configuration not only increases the *f*-number but also reduces specular reflection and lens crosstalk. The DF-LFC can provide high-contrast light-field images over a wide axial range, facilitating 3D *in vivo* imaging for clinical handheld operation [Fig. 1(c)].

## II. RESULT AND DISCUSSION

### A. Microfabrication and characterization of siMLAs

**FIG. 2.**
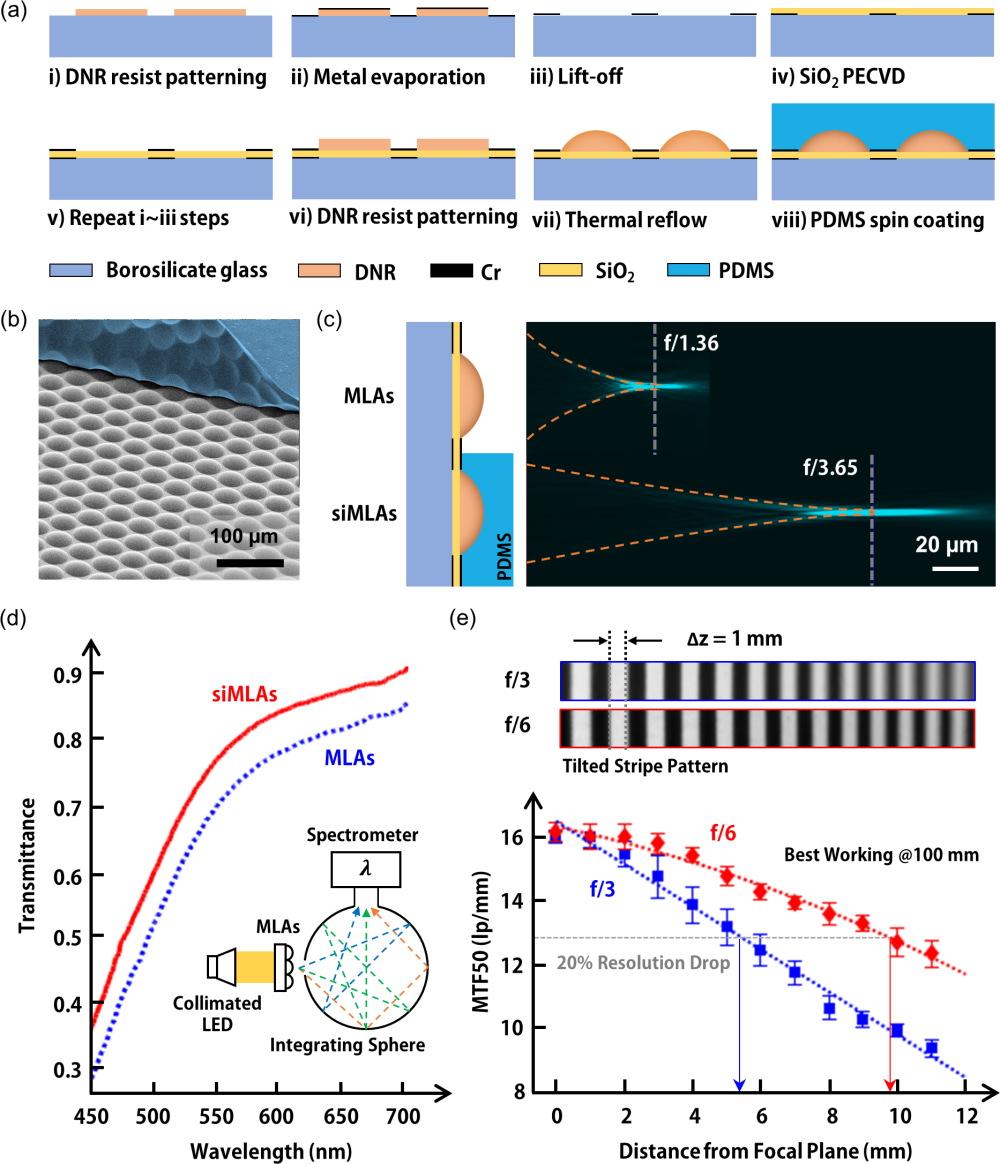
Microfabrication and optical properties of siMLAs. (a) The microfabrication steps of siMLAs including DNR-based MLAs and PDMS-based solid immersion medium. (b) A scanning electron microscopic image of siMLAs, where PDMS (pseudo blue color) on MLAs is partially peeled off. (c) Optical sectioning of the focused laser beam through microlenses before and after solid immersion. The *f*-number of MLAs is substantially increased by a factor of 2.7. (d) Comparison of optical transmittance between MLAs and siMLAs using a collimated white LED light source and an integrating sphere with a spectrophotometer. The transmittance increases by 5.6%–27% over the visible spectrum (450–700 nm) after solid immersion. (e) The reconstructed light-field of the striped pattern on a tilted plate and the MTF50 depending on object distance captured by DF-LFC prototypes with different *f*-number siMLAs. The siMLAs of *f*/6 exhibit approximately doubled DoF in the DF-LFC, compared to those of *f*/3.

The microfabricated siMLAs include a MIM absorber, resist-reflowed MLAs, and a low-index solid immersion medium [Fig. 2(a)]. First, a DNR resist (DNR-L300-D40, Dongjin Semichem Co., Ltd.) was photolithographically defined on a 4-in. borosilicate wafer (step i). A 5 nm thick chromium (Cr) layer was lifted off after e-beam evaporation (steps ii and iii). A 95 nm thick silicon dioxide (SiO_2_) layer was deposited by using plasma-enhanced chemical vapor deposition (PECVD) (step iv), followed by the liftoff of a 100 nm thick Cr layer to fabricate the MIM absorber (step v). The DNR resist was photolithographically defined (step vi) and thermally reflowed for the formation of MLAs (step vii). Finally, A 10 *μ*m thick PDMS (Sylgard 184, Dow, Inc.; 10:1 base-to-crosslinker ratio) layer was spin-coated on MLAs and cured at 120 °C for 20 min on a hot plate (step viii). The scanning electron micrograph shows that resist-reflowed MLAs are uniformly formed and completely immersed in thin PDMS film [Fig. 2(b)]. Note that the thin PDMS film was partially peeled off for the experimental demonstration.

Solid immersion increases the focal length and transmittance of MLAs in the experiments. The focal length of MLAs was measured by using a confocal laser scanning microscope (CLSM) with an additional collimating laser source (CPS532, Thorlabs, Inc.; 532 nm wavelength) [Fig. 2(c)].^26^ The *f*-number of MLAs increases by approximately 2.7 times after solid immersion in PDMS due to the refractive index change in the surrounding medium for microlenses. The experimental result is consistent with the calculated value and ray tracing [Fig. S1(a)]. The *f*-number of siMLAs is achieved up to *f*/6 using DNR-based MLAs and PDMS-based solid immersion medium [Fig. S1(b)]. The transmittance of MLAs and siMLAs was measured in the visible ranges (450–700 nm) using a collimated white LED light source (MCWHL2-C4, Thorlabs, Inc.), an integrating sphere, and a spectrometer (C10082CAH; Hamamatsu Photonics K.K.) [Fig. 2(d)]. The solid immersion of thin PDMS film increases the transmittance of microlenses by 5.6%–27% by reducing the index mismatch between the DNR resist and air. High transmittance of siMLAs further increases the image contrast, which apparently decreases the reconstruction errors in light-field imaging. Finally, DF-LFC prototypes were fully packaged with siMLAs of different *f*-numbers (*f*/3 and *f*/6) in order to reconstruct light-field images and measure MTF50 depending on the object distance [Fig. 2(e)]. Two different prototypes were designed for an object distance of 100 mm with a commercial main lens (C11–2520, Basler AG; 25 mm focal length). The light-field images of an axially tilted plate with a striped pattern were captured and reconstructed by using the digital refocusing algorithm to obtain wide-axial-range front views (see Sec. IV). In this experiment, the large DoF light-field image was captured using siMLAs of *f*/6, resulting in sharper line edges away from the focal plane [Fig. 2(e), top]. The DoF of the prototype with siMLAs of *f*/6 is roughly twice that with siMLAs of *f*/3 [Fig. 2(e), bottom], based on the MTF50 measurement (Fig. S2). These experimental results well match the light-field imaging theory, indicating that the DoF is approximately proportional to the *f*-number of MLAs.^10,22^ As a result, the large *f*-number of siMLAs allows substantially large DoF and high-contrast light-field imaging, unlike conventional LFCs.

### B. Light-field imaging results

**FIG. 3.**
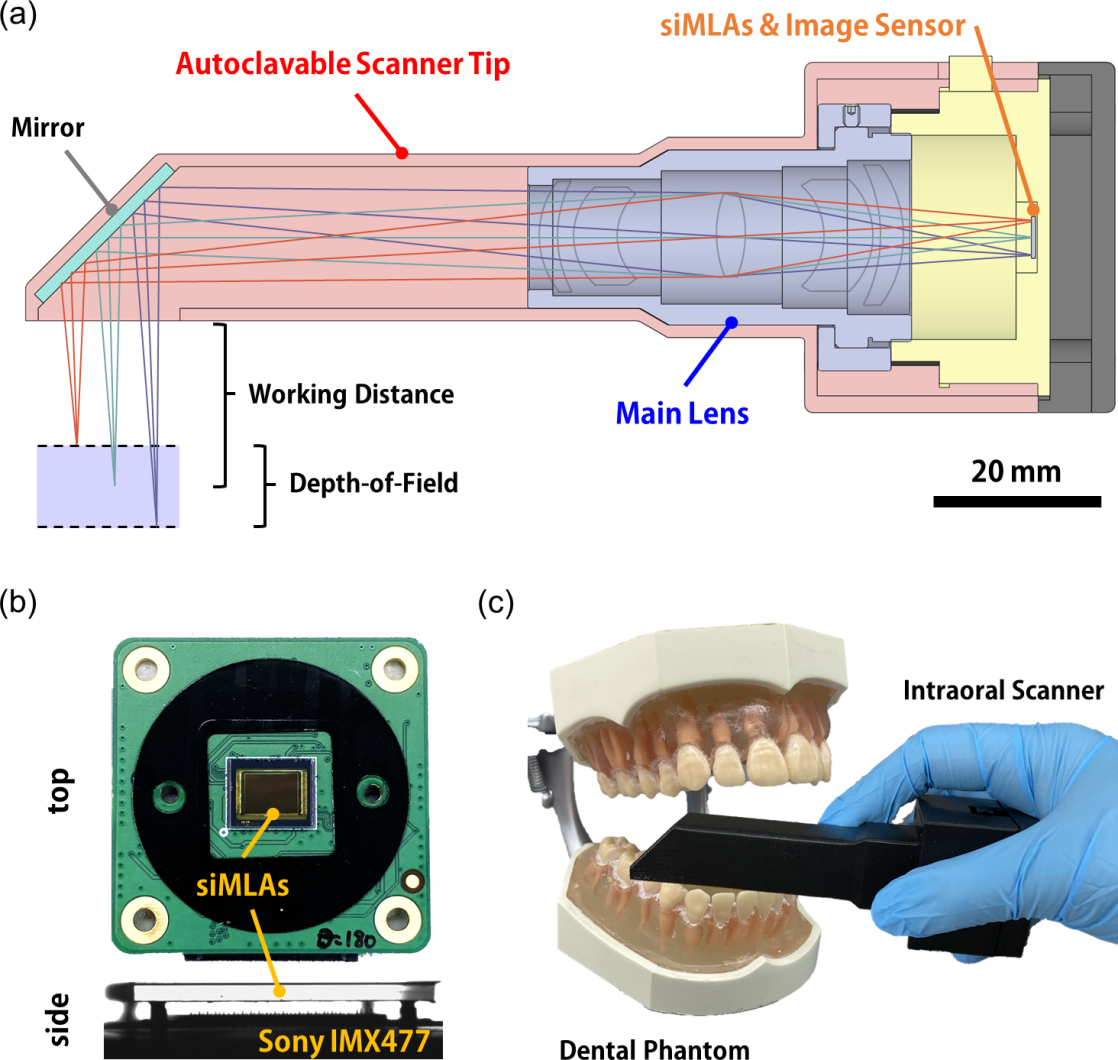
The fully packaged DF-LFC for handheld 3D intraoral scanning. (a) A schematic illustration of the DF-LFC based intraoral scanner, consisting of autoclavable scanner tip, main lens, and siMLAs on CMOS image sensor arrays. Photographs of (b) integrated siMLAs on CMOS image sensor arrays and (c) the packaged DF-LFC based intraoral scanner during handheld operation with an actual size dental phantom.

The DF-LFC was fully packaged for high-contrast and large DoF intraoral scanning, utilizing rapid prototyping. The intraoral scanner comprises a scanner tip, a custom-made main lens (28 mm focal length), siMLAs (*f*/3.6; 45 *μ*m pitch; hexagonal arrangement), and a single CMOS image sensor (IMX477, Sony Corp.; 12.3 *MP*, unit pixel: 1.55 × 1.55 *μ*m^2^) [Fig. 3(a)]. The scanner tip is removable for replacement and autoclaving after clinical use, and the planar mirror allows the scanning of inner teeth. The intraoral scanner has a working distance of 17–27 mm from the distal end of the scanner tip to facilitate focusing during the handheld operation inside the oral cavity. In addition, the intraoral scanner features a spatial resolution of less than 100 *μ*m to replace traditional dental impressions and a DoF of larger than 10 mm to ease handheld operation. See Sec. IV for the detailed design of DF-LFC. The siMLAs were integrated on a single CMOS image sensor where the curved surface of microlenses faces the image sensor with air gap spacing [Fig. 3(b)]. The intraoral scanner was connected to a single-board computer (Raspberry Pi 4 Model B) and the large volume light-field images were captured with an external illumination light source during the handheld operation [Fig. 3(c)]. The final 3D structure was reconstructed from the captured light-field images by using MATLAB (MathWorks, Inc.).

**FIG. 4.**
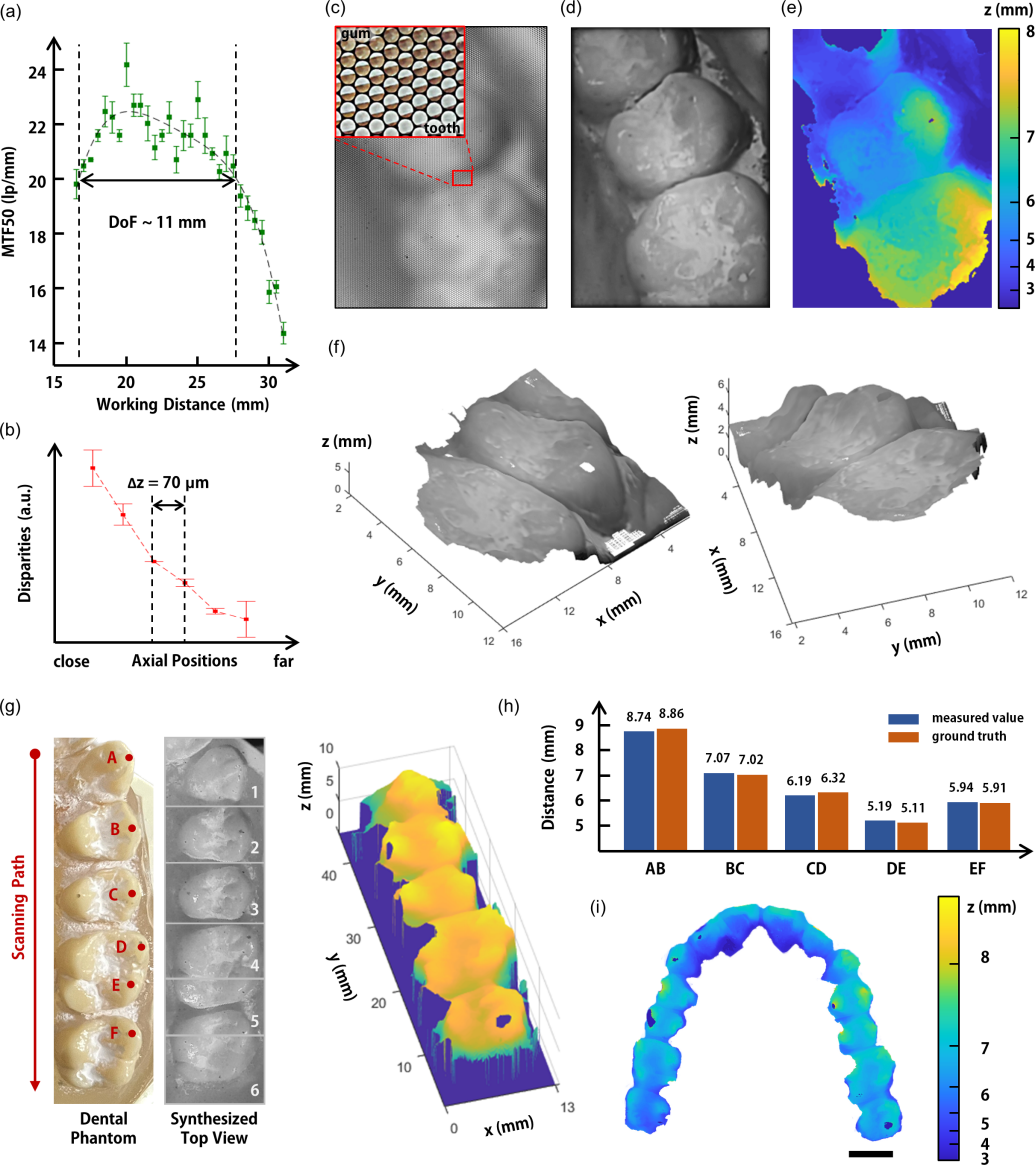
Light-field imaging using the fully packaged DF-LFC based intraoral scanner. (a) The measured MTF50 of the intraoral scanner. (b) The estimated axial resolution of the intraoral scanner by using a tilted plate with a line pattern. (c) The raw light-field image, (d) the synthesized top view, (e) the calculated depth map, and (f) the reconstructed multiview point-cloud images of a dental phantom captured from the intraoral scanner under a single exposure. (g) Stitched light-field images captured during intraoral scanning. Some cusp tips of the dental phantom are labeled A through F. (h) The quantitative comparison of the distances between dental landmarks in the reconstructed light-field and real geometry. The distances between neighboring cusp tips were measured using the intraoral scanner. (i) Reconstructed full-arch depth map of the dental phantom. The scale bar indicates 10 mm.

The volumetric imaging of a dental phantom has successfully been demonstrated by using the fully packaged intraoral scanner. The dental phantom may differ from the real oral cavity due to saliva and patient movement. However, saliva is often eliminated before scanning, and motion artifacts mainly result from handheld operations.^6^ The intraoral scanner exhibits MTF50 higher than 20 lp/mm for the axial range of around 10.5 mm, corresponding to the calculated DoF of 11.6 mm. [Figs. 4(a) and S2]. Note that the measured MTF50 decreases significantly after 10% drop (20.2 lp/mm) from the peak MTF50 (22.5 lp/mm). In addition, the intraoral scanner distinguishes the axial position differences of up to 70 *μ*m based on the disparity calculation of the line pattern on the axially tilted plate as aforementioned [Figs. 4(b) and S4]. The large DoF facilitates capturing raw images during the handheld operation without the use of a delicate optical stage due to the large DoF [Fig. 4(c)]. The magnified microimages (as shown in the inset) highlight the tooth-gum boundary with high contrast, and the formation of inverted images results from the Keplerian imaging scheme of the intraoral scanner. The 4D light-field data extracted from the raw image consist of angular (29 × 29) and spatial (272 × 193) information. The intraoral scanner acquires substantial volumetric data of 17 mm (W) × 13 mm (H) × 11 mm (D) with a single exposure by reconstructing the light-field data. An all-in-focus top view of the dental phantom was synthesized by stacking digitally refocused images with different focal planes [Fig. 4(d)]. The large DoF allows a single exposure to render a complete tooth in sharp focus. In succession, the depth map corresponding to the top view was computed by comparing synthetic images with different viewing angles using the cost volume-based stereo matching algorithm [Fig. 4(e)]. The depth map clearly shows the structural information such as the teeth groove, which is directly matched with the top view. The point-cloud images of the dental phantom were further reconstructed from the depth information. [Fig. 4(f), Movie S1]. In addition, multiple images were precisely stitched after intraoral scanning to obtain a panoramic light-field reconstruction of the dental phantom [Fig. 4(g)]. The distances between the cusp tips [labeled A–F in Fig. 4(g), left] of the dental phantom were measured to quantify the accuracy of the intraoral scanner [Fig. 4(h)]. The positions of the cusp tips were computed using the local maxima of the reconstructed volume. The ground truth data were obtained with a commercialized 3D structured light scanner (SmartScan 8MP, Hexagon AB; 4 kg weight; 240 mm stereo camera base length) with an accuracy of 10 *μ*m. The intraoral scanner measured the cusp tip distances with distance measurement errors of 27–133 *μ*m (mean: 84 *μ*m; standard deviation: 41 *μ*m), comparable to the conventional dental impression methods.^5,6^ A full-arch depth map was finally achieved by capturing 18 images [Fig. 4(i)].

## III. CONCLUSION

In summary, the DF-LFC has successfully been demonstrated for handheld 3D intraoral scanning using siMLAs. An additional low-index medium substantially increases the focal length and transmittance by reducing the index mismatch between MLAs and air. The siMLAs overcome the limited *f*-number range of conventional MLAs, facilitating the development of large DoF LFCs. The low specular reflection and MIM absorber of siMLAs also substantially improve the image contrast for accurate light-field reconstruction. The fully packaged DF-LFC driven intraoral scanner captures large volumetric information of a dental phantom under a single exposure during the handheld operation. DF-LFC based intraoral scanning allows not only precise 3D reconstruction with a simple configuration and single-shot large-volume imaging for easy handheld operation. In addition, it provides advanced digital impression capabilities, including high accuracy, reduced material waste, digital compatibility, and AI-assisted diagnosis, unlike conventional dental impressions. The DF-LFC can provide a new platform not only for intraoral scanning but also for assorted handheld 3D *in vivo* imaging systems in clinical endoscopy or robotic surgery.

## IV. METHODS

### A. Image reconstruction algorithms

The light-field data were obtained by using the Light-Field Imaging Toolkit (LFIT).^27^ The 4D light-field of a captured light containing the angular and spatial information was calculated by using the geometric parameters of the DF-LFC based intraoral scanner and microlens calibration. Figures 2(e) and 4(d) were also synthesized using the digital refocusing algorithm embedded in the LFIT. The digital refocusing algorithm was also used to reconstruct raw images of a slanted line pattern for the MTF measurement in Fig. 4(a). The cost volume-based stereo matching algorithm^28^ was used to calculate the slight differences in perspective-shifted views to obtain the disparity maps. The disparity comparison in Fig. 4(b) and the disparity map calculation of the dental phantom in Figs. 4(e), 4(g), and 4(i) were based on this algorithm.

### B. DF-LFC based intraoral scanner design

LFCs capture volumetric information about a scene with a single exposure by labeling the direction of light rays using MLAs. The working distance of intraoral scanners is about 20 mm from the distal end of the scanner tip, and the length of the tip is larger than 60 mm to facilitate scanning inner teeth efficiently. The optimal object distance of the DF-LFC is set to 80 mm to achieve the practical working distance and the scanner tip length by using a planar mirror at the end of the scanner tip [Fig. 3(a)]. The Keplerian-scheme LFC configuration was utilized for the intraoral scanner to obtain a large object distance and high resolution than standard and Galilean-scheme LFCs.^19^ The size of the mirror is 20.9 × 14.8 mm^2^ to cover the field-of-view (FoV) of up to 20 × 20 mm^2^ where the mirror is located at 50 mm from the main lens with 45° tilting. The scanner tip can be further minimized depending on the FoV design and the mirror size. The DoF and axial resolution of DF-LFC are determined by the relay of the main lens and MLAs. The DoF of MLAs plays a role in the depth-of-focus of the main lens, and the working range of the main lens conjugated with the DoF of MLAs determines the DoF of DF-LFC [Fig. S3(a)]. The large *f*-number of siMLAs increases the DoF of microlenses, thereby extending the DoF of DF-LFC. The DF-LFC based intraoral scanner has a theoretical DoF larger than 10 mm to achieve imaging of whole depth in a single frame, easy focusing without an electromechanical unit, and fast scanning from the large-volume acquisition. The theoretical DoF calculation was based on a geometric LFC model.^22^ The axial resolution was estimated using the axial position difference at the object space by single pixel deviation in the image plane. The intraoral scanner has a theoretical axial resolution of less than 100 *μ*m to achieve precise 3D reconstruction, comparable to traditional dental impression and conventional intraoral scanning. The more detailed specification of the intraoral scanner is summarized in the supplementary material.

## SUPPLEMENTARY MATERIAL

See the supplementary material for the additional fabrication result of siMLAs, the design of DF-LFC based intraoral scanner, the experimental setups of the results described in the main text, and the video showing the 3D reconstruction of the dental phantom.

## Data Availability

The data that support the findings of this study are available from the corresponding author upon reasonable request.
